# A superfamily of bioactive proteins from fungi - are these secondary metabolites?

**DOI:** 10.1186/s40694-026-00219-x

**Published:** 2026-08-29

**Authors:** Claudia Feurstein, Lisa Bachmann, Lena Heber, Birgit Dobbert, Sascha Jung

**Affiliations:** Chair of Applied and Molecular Microbiology, Technische Universität Berlin, Straße des 17. Juni 135, 10623 Berlin, Germany

**Keywords:** Antifungal protein, Bubble protein, NFAP2, Gamma-core, Filamentous fungi, BLAST, Phylogenetic analysis, Secondary metabolites

## Abstract

**Background:**

Since decades, fungi are leveraged in biotechnology to produce high-value compounds used in multiple economic sectors. Strain and process optimisation is based on a comprehensive understanding of the production organism on the cellular and molecular level. Among three antifungal protein families in fungi, consisting of small cysteine-stabilised proteins, it has been shown that, for some family members, bioactivities are also associated with additional functions in their hosts, e.g., carbon metabolism, autophagy, or asexual development. These proteins are interesting as alternative source of novel antifungal drugs. However, their potential impact on biotechnological production is not yet elucidated.

**Results:**

In this study, we introduce the antifungal bubble protein “AgBP”, from *Aspergillus giganteus* and further elucidate the reservoir of bioactive proteins in fungi. We used NCBI PSI-BLAST and subsequent phylogenetic and structural analyses of the Antifungal Protein (AFP), Bubble Protein (BP), and *Neosartorya fischeri* antifungal protein 2 (NFAP2) family members. We could identify further putative members: 165 AFP-, 102 BP-, and 219 NFAP2-like proteins. Six of the AFP and all 219 NFAP2 family members are not yet assigned on InterPro. All proteins were exclusively identified in fungi. To our best knowledge, this is the first study to report this group of bioactive proteins is shared among the two divisions of Ascomycetes and Basidiomycetes. Phylogenetic tree analyses demonstrate restricted taxonomic distribution within single genera. Furthermore, the comparison of the tertiary structures of all members of the three AFP families clearly separates them from each other and from non-fungal small cysteine-stabilised antifungal proteins.

**Conclusion:**

We hypothesise that the three protein families represent a distinct superfamily of evolutionary related proteins. We further hypothesise that these proteins could be categorised as secondary metabolite like molecules of ribosomal origin. For brevity, we named this superfamily BPF (bioactive proteins from fungi). The distribution of BPF members is presumably driven by horizontal gene transfer. Furthermore, we hypothesise that BPF members likely serve rather different biological roles than merely acting as antimicrobials. Their hypothetical classification as potential secondary metabolite like proteins in combination with their occurrence among several biotechnologically relevant fungal genera, e.g., *Aspergillus*, *Penicillium*, *Trichoderma*, *Schizophyllum*, etc., emphasises their potential relevance for genetic and metabolic engineering.

**Supplementary Information:**

The online version contains supplementary material available at https://doi.org/10.1186/s40694-026-00219-x.

## Background

Fungi have become indispensable in biotechnology. They are leveraged to produce a broad set of economically relevant compounds, e.g., organic acids, platform chemicals, enzymes, and therapeutics [1]. Their exploitation is based on a comprehensive understanding of their biology which has not yet been elucidated in full detail.

Antifungal proteins from Ascomycetes have been identified to have exogenous and endogenous functions within the fungal cell [2, 3]. On the one hand, they exhibit antimicrobial activity against a range of organisms, such as filamentous fungi, yeast, bacteria, and/or viruses depending on the tested protein and conditions [4, 5]. However, they rarely show phytotoxic or cytotoxic effects [6–10], which may be explained by their specific modes of action. Different models are described including binding of fungal specific cell wall compounds like chitin, affecting specific chitin synthases, activation of the CWI pathway, membrane lipid binding and membrane permeabilisation, dependence on glycosyl desaturases, and/or triggering calcium homeostasis by activating the cAMP/PKA pathway [3, 11–13].

On the other hand, the endogenous function of some antifungal proteins has been shown to drive vegetative growth and nutrient mobilisation [2, 3]. Expression of *Aspergillus niger* Antifungal Protein (AnAFP) in *A. niger* induces autophagy and impacts other biological processes, e.g., cell wall remodelling, carbohydrate metabolism, and cellular transport processes [2]. Furthermore, AnAFP is considered as an important factor in asexual sporulation whose expression (transcription and translation) is strictly controlled in time and space.

The physiochemical features of antifungal proteins found in filamentous fungi categorise them into the group of antimicrobial peptides: they are small, have a positive net charge, and an amphipathic character [5, 14]. Antifungal proteins produced by fungi are classified differently in literature, e.g., as either different classes, or as different families, or as different classes of one family, and individual groups are even referred to as superfamily [5, 15, 16]. Based on their primary and tertiary structures, they show distinct differences [5]. All antifungal proteins produced by fungi contain a γ-core motif, which consists of two beta strands and is primarily identified in antimicrobial proteins [17, 18]. This motif appears in three isoforms called dextromeric (NH_2_⋅⋅⋅[X_1–3_]-[GXC]-[X_3–9_]-[C]⋅⋅⋅COOH), levomeric 1 (NH_2_⋅⋅⋅[C]-[X_3–9_]-[CXG]-[X_1–3_] ⋅⋅COOH), and levomeric 2 (NH_2_⋅⋅⋅[C]-[X_3–9_]-[GXC]-[X_1–3_] ⋅⋅COOH) [17, 18].

**Fig. 1:**
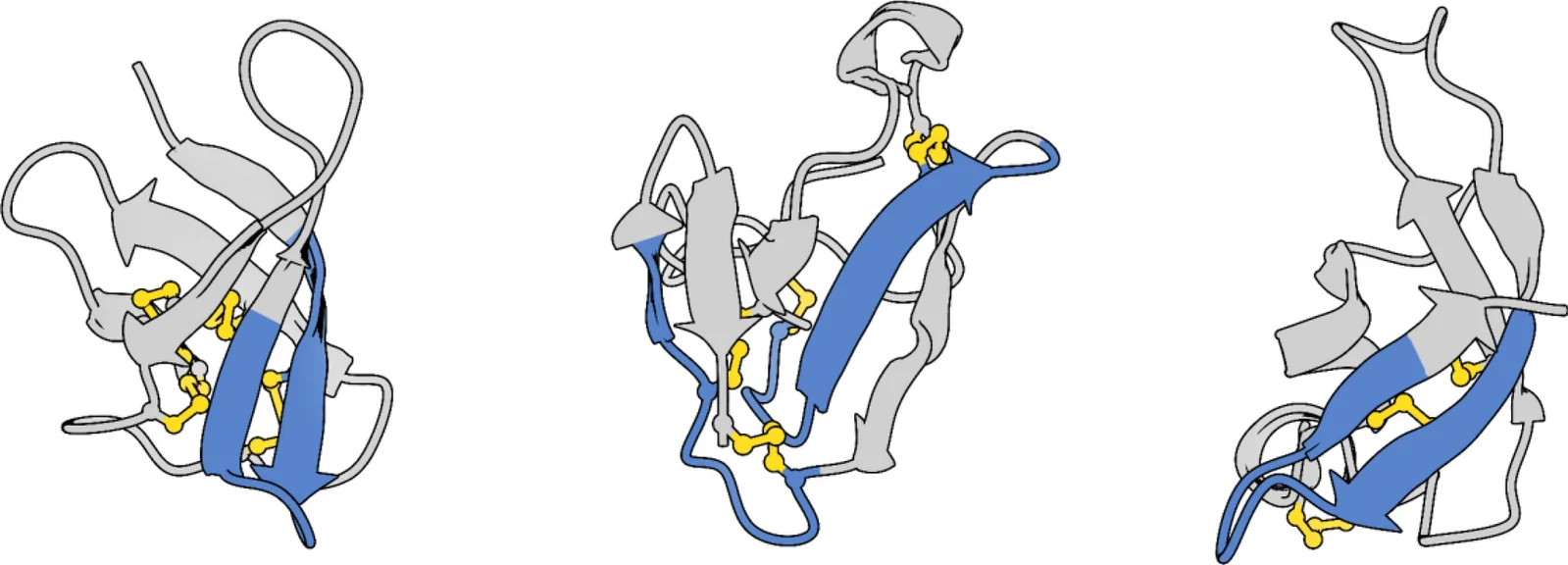
Three-dimensional structures of the founding proteins of the three cysteine-stabilised antifungal protein families in fungi: AgAFP, PbBP, and NFAP2. Structure visualisation as ribbon model of AgAFP from *A. giganteus* (Protein Data Bank file (PDB): 1afp Model 21; left), PbBP from *P. brevicompactum* (PDB: 1uoy Model 1; middle), and NFAP2 from *N. fischeri* [new name, *A. fischeri*] (PDB: 8rp9 Model 1; right) was performed in ChimeraX. Disulfide bridge forming cysteines are highlighted in yellow and the γ-core motifs are indicated in blue

**Fig. 2:**
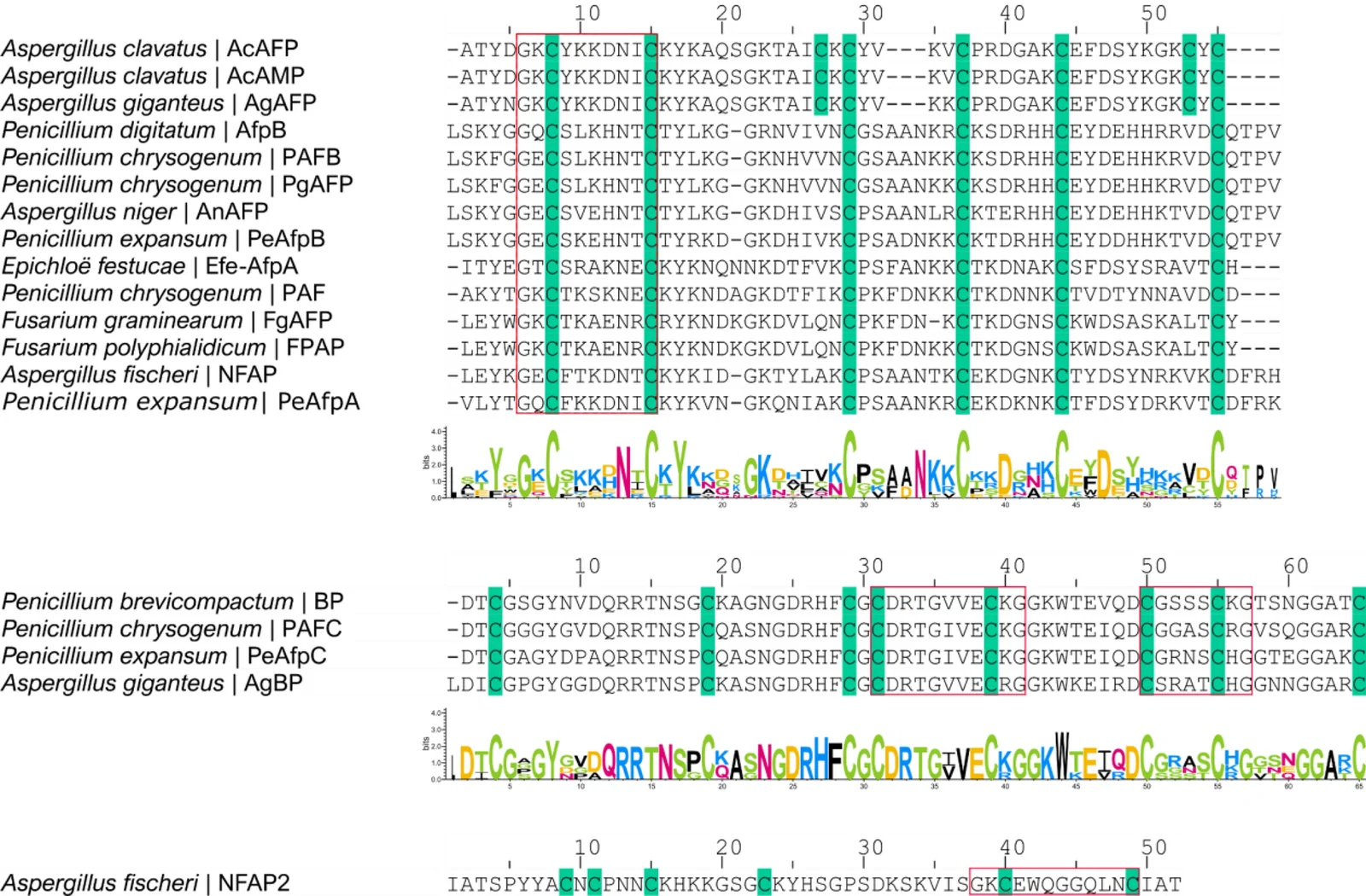
Multiple sequence alignments of the mature regions of antifungal proteins from fungi show distinct structural differences in the protein primary structure. Multiple sequence alignment was performed and sequence logos generated using the Multiple Alignment using Fast Fourier Transform (MAFFT) algorithm and WebLogo3 for published verified members of the AFP (top) and the BP (middle) family [28–31]. Red squares indicate the position of the γ-core motif. Names of the fungal hosts and of the proteins are listed on the left. Note, during the final phase of the current study a new member of the BP family from *A. fischeri* (AfAfpC) was described by Capra et al. [32], after major analyses of the current study has been performed. Since the primary structure is highly similar to the four BP, it has no impact on the current study

Olsen and Goerner were the first to describe the antifungal protein “AFP” from *Aspergillus giganteus* in 1965 [19]. For clarity reasons, the “AFP” from *A. giganteus* will be denoted as “AgAFP” in the following text. AgAFP is the founding molecule for the first defined family of antifungal proteins from filamentous fungi: the Antifungal Protein (AFP) family [3]. This protein is small (5.8 kDa), positively charged, amphipathic, and features a beta barrel structure with five beta strands stabilised by four disulfide bridges [20]. Six of its eight cysteines are conserved among all members of the AFP family, which contain a dextromeric γ-core motif at the N-terminal moiety and an average length of 92 amino acids in the precursor protein (Figs. 1 and 2). A second protein family which is related to the AFP family is formed by the bubble protein-like proteins, named after the Bubble Protein (PbBP) from *Penicillium brevicompactum* described by Olsen et al. in 2003, which was identified in secreted droplets on fungal mycelium [21]. Bubble Protein (BP) family precursor proteins are on average 93 amino acid long and the mature proteins adopt a knottin-fold, stabilised by four disulfide bridges [15]. All eight cysteine residues are conserved among family members which contain two levomeric 1 γ-core motifs in their primary structure (Figs. 1 and 2; [15]). A third family of antifungal proteins is named after *Neosartorya fischeri* antifungal protein 2 (NFAP2) from *N. fischeri* [new name, *Aspergillus fischeri*], discovered in 2016 and the up to date only verified member of this family [22]. The NFAP2 precursor is 86 amino acids long and the mature protein contains three disulfide bridges and a dextromeric γ-core motif in the C-terminal moiety [22, 23]. Due to the low number of described members in the NFAP2 family a further distinction is not yet possible (Figs. 1 and 2).

Evolutionary investigations of antifungal proteins from fungi can extend the conclusions drawn from protein structure and physiochemical properties. Phylogenetic analysis of pre-pro-proteins gives valuable insight into the evolution of, e.g., secretory pathways, activation mechanisms, or horizontal gene transfer (HGT; [24, 25]). Pre-pro-proteins differ in their evolutionary rate from the evolution of the mature proteins, whose phylogenetic analysis provides insights in the progression of protein function and diversification restricted by their function [26, 27].

In this study, we isolated and investigated a new antifungal protein of *A. giganteus* which belongs to the BP family. Furthermore, we investigated the relationship between three families of antifungal proteins from fungi by detecting putative new members and their respective encoding organisms using the Position Specific Iteration - Basic Local Alignment Search Tool (PSI-BLAST) and phylogenetic tools. We conclude that the AFP, the BP, and the NFAP2 family members might be propagated via HGT, are exclusively encoded by fungi, should be referred to as secondary metabolite like proteins, and represent a superfamily distributed among two divisions of fungi. Recognising AFP, BP, and NFAP2 family members as potential secondary metabolites, would enable a deeper understanding of their function and thus might impact metabolic engineering in biotechnology.

## Methods

### BPF families’ dataset acquirement

The workflows described below are as described elsewhere [33].

Further hypothetical members of AFP, BP, and NFAP2 families were identified with PSI-BLAST (https://blast.ncbi.nlm.nih.gov/Blast.cgi) from the National Center for Biotechnology Information (NCBI) (blastp with PSI-BLAST; accessed: 16.07.2025). The pre-pro-protein sequences of the UniProt accession numbers P17737 (AgAFP from *A. giganteus*), G5DC88 (PbBP from *P. brevicompactum*), and A1DBL3 (NFAP2 from *N. fischeri* [new name, *A. fischeri*]) were used as query sequences. Filter characteristics given in Table 1 were concluded from analyses of the structural properties of verified proteins shown in Fig. 2 and applied after each iteration. The sequence length used to search for unknown members of the different families was defined by multiplying the average length concluded from verified members (Fig. 2) with a factor of 1.5 giving the lengths stated in Table 1. Protein sequences exceeding 1.5 times the average length of the verified pre-pro-proteins were excluded. Thus, unknown family members which might exceed the length of the known members can still be identified, whereas coincidental matches with unrelated proteins are limited. Proteins without information for pre-pro-sequences were also excluded to form the Position Specific Scoring Matrix (PSSM) for the next iteration. The occurrence of the γ-core motif was assessed through the ProSite tool [34].

**Table 1:** Minimal search criteria set for respective protein family

	AFP-like	BP-like	NFAP2-like
Length in amino acids in precursor protein	141	146	129
γ-core motif	< x(1,500)-G-x-C-x(3,9)-C	C-x(3,9)-C-x-G-x(1,500)-C-x(3,9)-C-x-G-x(1,500) >	< x(1,500)-G-x-C-x(3,9)-C
Number of cysteines in mature protein	≥ 6	≥ 8	≥ 6

Numbers in brackets define the minimum (first position) or maximum number (second position) of residues adjacent to defined amino acids of the γ-core motif, i.e., cysteine and/or glycine. “x” is an arbitrary proteinogenic amino acid. The minimum number of cysteines per family was 6 or 8, respectively

Multiple Alignment using Fast Fourier Transform (MAFFT) algorithm version 7 was used to form the alignment of all proteins of each family (List of precursor and putative mature proteins Supplementary File 1; Multiple sequence alignment Supplementary Fig. 1; [30, 31, 35]). Sequence logos were analysed through WebLogo version 3.9.8 [28, 29]. The position of the putative first amino acid of mature proteins was concluded from the alignment of all found proteins to the respective query sequence (indicated in Supplementary Fig. 1). Residues on the N-terminal site of the mature query protein were considered as pre-pro sequences and removed for respective analysis. Furthermore, proteins with more than ten residues in front of the first conserved cysteine were excluded from analysis (relevant for 26 proteins) to minimize alignment errors [36]. The length of 10 residues was concluded from the alignment shown in Fig. 2. None of the verified proteins had more than 10 residues in front of their first conserved cysteine residue. Maximum-likelihood analysis of putative mature proteins was performed using IQTree online tool with a bootstrap of 1,000 [37] and the Defensin-like protein 2 (RsAFP2, NCBI ID: XP_018461236.1) from *Raphanus sativus* was used for outgroup rooting. The plant-derived defensin was selected because, although it is not of fungal origin, it exhibits similar properties in terms of length, cysteine content, and antifungal activity. This is consistent with earlier studies [16].

To build species trees, organism strains and their respective GenBank Assembly (GCA) numbers were retrieved using NCBI Dataset command line tools (CLIs; [38]). Supplementary Files 2 and 3 list the GenBank assemblies used for the protein-encoding species tree and the phylogenetic tree of taxonomic distribution, respectively. Trees were build using the Universal Fungal Core Genes (UFCG) pipeline v1.0.6 incorporating the FastTree module with default settings [39]. For outgroup rooting the genome of *Saccharomyces cerevisiae* (GCA_000146045.2) was used. Organisms not included due to lack of any genome assembly: *A. giganteus* and *Aspergillus spathulatus.* Reference genomes were used when a strain-specific genome assembly was unavailable or the strain was not specified in the protein description. All phylogenetic trees were visualised using iTOL v5 [40]. The database InterPro (https://www.ebi.ac.uk/interpro/) was used to screen output proteins assignment to a protein family, using protein sequences (Antifungal Protein: IPR022706; Bubble Protein: IPR015308; NFAP2: -; accessed: 20.08.2025).

### Tertiary structure comparison of antifungal proteins

Tertiary structure similarity comparison was performed using USalign (Version 20,241,108) [41]. PDB files were retrieved by UniProt ID mapping or AlphaFold2 v1.5.5 batch prediction of the precursor sequences, using settings to maximise accuracy while limiting computational time (Table 2). Subsequent trimming of the top-ranking model to the putative mature sequence was performed using ChimeraX version 1.10 [42–44]. Structures available in the PDB database were simplified by retaining only their first model and removing heteroatoms (e.g., ligands or ions). A comprehensive list of UniProt accession numbers or PDB identifiers is available in Supplementary File 4 and of non-fungal cysteine stabilised antifungal proteins (precursor protein length ≤ 150 amino acids) in Supplementary File 5. Classical multidimensional scaling (MDS) using RStudio’s cmdscale package converted the distance (1-TMscore) matrix to two-dimensional coordinates [45–47].

**Table 2:** AlphaFold2 batch settings

Msa_mode	MMseqs2 (UniRef + Environmental)
Num_models	5
Num_recycles	12
Num_relax	1
Relax_max iterations	200

### Cultivation of *A. giganteus* and purification of AgBP

*A. giganteus* spores (2 × 10^8^) were incubated in 500 mL Erlenmeyer flasks containing 200 mL complex medium (1% glucose, 0.1% casamino acids, 0.5% yeast extract, 2 mM MgSO_4_xH_2_O, 70 mM NaNO_3_, 11 mM KH_2_PO_4_, 7 mM KCl, 0.1% modified Vishniac trace element stock solution [48]) at 30 °C and 150 rpm for 92 h. Biomass was removed by filtration and subsequent centrifugation (3,090×g, 10 min). The clear supernatant was further purified by hydrophobicity chromatography (Sep-Pak, 6 cc tC18, Waters Corporation). The column was washed with 84% acetonitrile acidified with 0.1% trifluoroacetic acid (TFA) and equilibrated with 0.1% TFA acidified water before supernatant was applied. Non-specifically bound compounds were washed out with 0.1% TFA acidified deionised water. Bound sample was eluted in 20% acetonitrile acidified with 0.1% TFA. The elution sample was diluted 1:3 with phosphate buffered saline and applied to cation chromatography using a Toyopearl Sulfate-650F SkillPak 5 mL column (TOSOH Bioscience) equilibrated in 50 mM sodium acetate, pH 5. Using continuous gradient fast performance liquid chromatography (1 mL min^−1^ flowrate) bound compounds were eluted at increasing concentrations of sodium chloride by addition of 50 mM sodium acetated, pH 5, supplemented with 1 M NaCl (2% min^−1^) from zero to 100%. Fractions of interest were further purified by reverse phase (RP) HPLC using a gradient approach at a flowrate of 1 mL min^−1^ and a C8 column (Multo High—Bio 200–5, 200 mm × 5 mm; Chromatographie Service GmbH, Germany) starting at 2% acetonitrile acidified with 0.1% TFA. Acetonitrile concentration increased by 3.1% min^−1^. Fractions of interest showing single peak purity were concentrated (Amicon® ultra filter, 3 kDa, Merck KGaA, Germany). Acetonitrile was removed using vacuum centrifugation. The remaining aqueous solvent was removed by freeze drying. The freeze-dried samples were reconstituted in 0.01% TFA for further investigation.

### Protein fingerprint analysis for identification of the mature bubble protein from *A. giganteus* and gene identification

A 10 µg protein sample (1 µg µL^−1^ in 0.01% TFA) was diluted with 40 µL denaturing buffer (8 M urea, 50 mM ammonium bicarbonate). Disulfide bridges were reduced with 10 µL 50 mM tris(2-carboxyethyl)phosphine (TCEP) for 30 min at room temperature, then alkylated with 10 µL 55 mM iodoacetic acid in the dark for 20 min at room temperature. Proteins were digested using 0.5 µg trypsin (1 µg µL^−1^ in 0.1% TFA) for around 15 h at room temperature, desalted with C18 StageTips, and eluted with 40 µL 80% acetonitrile/0.1% TFA. Afterwards, the sample was dried by vacuum centrifugation and resuspended in injecting buffer (0.1% TFA, 1.6% acetonitrile) to 0.14 µg µL^−1^. For LC–MS/MS, injections included 50 ng and 500 ng samples. LC–MS/MS was conducted using an Orbitrap Fusion Lumos Tribrid mass spectrometer with an Ultimate 3000 RSLCnano System (Thermo Fisher Scientific). Proteins were separated at 50 °C on a 50 cm EASY-Spray C18 column. Mobile phase A and B consisted of water and 80% acetonitrile, respectively, both containing 0.1% formic acids. Proteins were loaded and eluted at 0.3 μL/min, using a gradient from 2 to 35% B over 35 min, then to 40% B in 5 min, and finally to 90% B in 1 min. Ionisation was performed via an EASY-Spray source (Thermo Scientific). MS data were acquired in data-dependent, top-speed mode. Each three-second cycle included a full scan in the Orbitrap at a resolution of 120,000. Ions with charge states 2 + to 7 + were selected and fragmented by higher-energy collisional dissociation (collision energy 30%), with fragmentation spectra recorded in the Orbitrap at a resolution of 50,000. Dynamic exclusion was set to single repeat count and 30 sec exclusion duration. Protein identification was performed with MaxQuant (v1.6.3.4) using default settings. MS/MS peak lists were searched against other bubble protein-like sequences [16].

Genome sequencing data from *A. giganteus* strain CBS 515.65 deposited at the Joint Genome Institute (JGI) genome portal (Project ID: 1051940) was used to identify the AgBP gene. The protein fragment “HFCGCDR” identified by MS/MS was used to search *A. giganteus* genome contigs, revealing the AgBP amino acid sequence. Using the corresponding identifier, Aspgig1 GeneModels (FilteredModels) were screened which led to the identification of the *agbp* gene.

### Isolation of genomic DNA and sequencing

Strains IfGB15/0901, /0902, /0903, and /0904 of *A. giganteus* were cultivated and biomass was prepared and lysed in 500 µL DNA extraction buffer as described before [50]. The DNA purification was done without the usage of phenol–chloroform-based phase separation. Instead, 2 µg RNase was added to the lysate, incubated for 15 min at 65 °C. After cooling the samples on ice for 5 min, 100 µL 8 M potassium acetate was used to precipitate proteins/lipids by centrifugation (10,000×g, 10 min). The supernatant was transferred into reaction tubes for DNA precipitation by addition of 300 µL isopropanol and centrifugation (10,000×g, 10 min). The DNA pellet was washed in 500 µL 70% ethanol and centrifuged (10,000 × g, 10 min). After decanting the supernatant residual ethanol was removed by evaporation. Finally, the DNA was resuspended in 50 µL water.

Primers were designed based on the annotations of the reference-genome for *A. giganteus* strain CBS 515.65 deposited at the JGI genome portal (Project ID: 1,051,940) using scaffold 18 for AgAFP and 69 for AgBP; Supplementary file 6). AgAFP and AgBP loci were amplified from genomic DNA by PCR using Phusion TM High-Fidelity DNA Polymerase (Thermo Fisher Scientific Inc., USA) and primer pairs 4984 and 4985 or 4925 and 4926, respectively. PCR products were purified through innuPrep DOUBLEpure Kit (AJ Innuscreen GmbH, Germany) and sequenced in two reactions with 4861 or 4862 for AgBP gene and with 4927 or 4928 for AgAFP gene by LGC Genomics GmbH, Berlin, Germany.

### Bioactivity assays

A microtiter plate assay measured the growth inhibition of AgBP on various test organisms, including filamentous fungi (*Aspergillus niger*, *Penicillium chrysogenum, Fusarium sporotrichioides,*), yeast (*Pichia pastoris* [new name: *Komagataella phaffii*], *Saccharomyces cerevisiae*, *Candida albicans*), and bacteria (*Escherichia coli*, *Bacillus subtilis*, *Enterococcus* sp.). Each well contained 1 × 10^3^ spores or cells in 100 µL of two-fold concentrated yeast extract peptone dextrose (YPD) medium (pH 6 was used for yeast, and pH 4.5 for filamentous fungi and bacteria). YPD contained 1% yeast extract (Ohly), 2% peptone (Gibco Bacto), and 2% glucose. Per well, 100 µL of AgBP in 0.01% TFA acidified water were added to final concentrations ranging from 0.4 to 50 µg mL^−1^. After 48 h at either 30 °C for fungi and yeast or 37 °C for bacteria, optical density was measured at 600 nm. A mixture of 100 µL YPD medium and 100 µL 0.01% TFA acidified water was used as blank. For positive and negative controls, spores or cells were incubated with or without 2% sodium dodecyl sulfate dissolved in 100 µL YPD medium and 100 µL 0.01% TFA acidified water, respectively. For each test organism two independent experiments have been conducted as quintuplicate. The Minimal Inhibitory Concentration (MIC) for the experimental setup used in this study was defined as the protein concentration leading to a growth inhibition of at least 90% compared to untreated controls.

### Use of artificial intelligence

ChatGPT 5 (version 5.1, and 5.2, OpenAI) was used as interactive search engine only, but not for drafting or writing the manuscript or parts of it.

## Results

### *Aspergillus giganteus* expresses the BP family member AgBP in parallel to AgAFP

Intriguingly, we could isolate a previously uncharacterised antifungal protein from *A. giganteus* cultivation medium, which was isolated additionally to AgAFP when modified isolation protocols have been tested to further optimise the downstream processing for AgAFP. Based on the cationic character of AFP, BP, and NFAP2 homologous proteins, ion chromatography is frequently used during their purification [8, 32, 51]. In contrast to routine purification methods applied in our laboratory comprising chitin-affinity chromatography [13, 52, 53], the application of hydrophobic interaction chromatography on culture supernatants enriched a protein which showed a band shift on discontinuous sodium dodecyl sulfate polyacrylamide gels compared to an AgAFP control sample (Supplementary Fig. 9A). The corresponding elution fraction also contained AgAFP, albeit to a much lesser extent (Supplementary Figs. 9B and 9C). During MS/MS analyses three peptides matching primary structures of a list containing BP family members were identified, which included the sequence “HFCGCDR” (950.3487 Da). Latter is highly conserved in the BP family (Fig. 2 and [32]).

The protein fragment “HFCGCDR” identified by MS/MS was used to search *A. giganteus* genome contigs, revealing the sequence *MKITALLYTLMAVATVSASA*↓LPQGDFQVLDICGPGYGGDQRRTNSPCKASNGDR**HFCGCDR**TGVVECRGGKWKEIRDCSRATCHGGNNGGARC, (Aspgig1|327533). The sequence shown in italics is the SignalP-4.0 [49] predicted signal protein (arrow indicates cleavage site), the underlined portion matches the monoisotopic mass (6905 Da) of the isolated protein from *A. giganteus* cultivation medium, and the bold segment represents the protein from fingerprint analysis. The eight amino acid residues in between the predicted signal peptide and the verified mature region might point to further processing steps and a large form AgBP (lf-AgBP) which could not be isolated yet. The mature protein was denoted as “AgBP” (the predicted tertiary structure is depicted in Supplementary Fig. 2; a sequential alignment with other verified BP members is depicted in Fig. 2).

According to the high similarity of the primary structures among BP members (Fig. 2), the predicted AgBP model structure is similar to the tertiary structure of other bubble protein-like proteins. It shows a knottin-fold with the four disulfide bridges located in its inner part (compared with representative structure of PbBP in Fig. 1). This is consistent with other studies [32]. The corresponding gene encoding AgBP was found on scaffold_69 (JGI coding sequence (CDS) between the start codon at 39,283 and the stop codon at 39,628). As other BP genes [54], the *agbp* gene contains one intron and was 100% conserved among genomes of five different *A. giganteus* strains tested in this study (data not shown). In contrast, the *agafp* gene showed several deviations on the nucleic acid and amino acid level indicating a different mutational rate for both genes (Supplementary File 7).

Based on the initial antimicrobial activity screening, including representative for fungi, bacteria and yeast, AgBP exerted a relative narrow growth inhibitory activity spectrum including only a few species of filamentous fungi, e.g., *Aspergillus niger* (Minimal Inhibitory Concentration (MIC) ~ 50 µg mL^−1^), *Fusarium sporotrichioides* (MIC ~ 6.25 µg mL^−1^), and yeast, e.g., *Pichia pastoris* [new name: *Komagataella phaffii*] (MIC ~ 1.6 µg mL^−1^). The growth of bacteria, e.g., *Escherichia coli*, *Bacillus subtilis*, *Enterococcus* sp., and further strains from yeast, e.g., *Saccharomyces cerevisiae*, *Candida albicans*, or filamentous fungi, e.g., *P. chrysogenum*, was not inhibited at concentrations of up to 100 µg mL^−1^ of AgBP (Supplementary Fig. 3 and data not shown). Interestingly, there is a strong paradoxical effect with *F. sporotrichioides* which can be also detected for *P. pastoris* [new name: *Komagataella phaffii*], albeit to a much lesser extent.

### Putative members of the AFP, BP, and NFAP2 family are encoded by Ascomycetes and Basidiomycetes and show a diverse evolutionary history

A series of *in silico* analyses were conducted to examine the phylogenetic relationships among the AFP, BP, and NFAP2 protein families. Utilising the NCBI PSI-BLAST and applying the specified filter criteria (Table 1), a total of 165 AFP, 102 BP, and 219 NFAP2 homologues were found after multiple iterations. Of these, six AFP family members were possible additional members of the corresponding protein family on InterPro, while all BP family members have been grouped into the BP family. In contrast, no NFAP2 family member was so far assigned to a protein family on InterPro, as this family has yet to be formally defined.

Interestingly, all identified 486 members of the three families (distinguished by characteristic primary structures and protein folds) are exclusively encoded by fungi and might apparently form a superfamily of evolutionary related proteins, which we denoted as BPF (*bioactive proteins from fungi*) and which must be demarcated from the recently defined group of “Fungal-Derived Bioactive Peptides” [55]. In contrast to BPF, Fungal-Derived Bioactive Peptides comprise peptides consisting of 2–30 residues, their molecular weight is below 5 kDa, and they were derived by treatment of various food sources with fungal strains to release those peptides by fermentation processes [55]. Note, regarding BPF, the term “superfamily” is primarily referring to the phylogenetic relation based on structural characteristics (see Table 1 and Fig. 2) and origin (fungi) whereas functional aspects must be considered more carefully, as will be discussed later. The classes Eurotiomycetes (35.6%) and Sordariomycetes (46.5%) within the phylum Ascomycetes, and the class Agaricomycetes (14.0%) within the phylum Basidiomycetes are the most abundant organism classes encoding BPF (Table 3). Yeast strains have not yet been identified among the encoding fungal organisms.

**Table 3:** Distribution of antifungal proteins from AFP, BP, and NFAP2 families among fungal species

Phylum/Class	AFP family	BP family	NFAP2 family	Sum	Sum [%]
**Ascomycetes**					
Eurotiomycetes	108	58	7	173	35.6
Sordariomycetes	51	42	133	226	46.5
Dothideomycetes	5	2	6	13	2.7
Leotiomycetes	1	0	3	4	0.8
Candelariomycetes	0	0	2	2	0.4
**Basidiomycetes**					
Agaricomycetes	0	0	68	68	14.0
Sum	165	102	219	486	

Zooming into genus level, the three major BPF encoding fungi are *Aspergillus* (18.3%), *Penicillium* (15.8%), and *Claviceps* (10.1%, Supplementary File 8). Whereas *Aspergillus* (AFP-like: 35.2%; BP-like: 23.5%) and *Penicillium* (AFP-like: 26.1%; BP-like: 33.3%) are the primary genera of AFP and BP family member encoding fungi, the main genera encoding NFAP2 family members are *Claviceps* (22.4%) and *Hypoxylon* (9.6%). In contrast, the genus *Aspergillus* accounts for only 3.2% and the genus *Penicillium* encodes no NFAP2 family member. This clear separation hints towards a complex development of BPF superfamily members.

Comparing the primary structure characteristics molecular weight (kDa), sequence length, charge at pH 7.4, “Grand Average of Hydropathy” (GRAVY), and amino acid composition of the predicted mature proteins between AFP, BP, and NFAP2 families, only a few amino acids show a similar distribution (Supplementary Fig. 4). In particular, a higher glycine, arginine, aspartate, glutamine content is characteristic for BP family members, whereas lysine is more frequent in AFP family members. NFAP2 family members are characterised by an increased hydrophobicity (GRAVY), but a decrease of both length and molecular weight in comparison to the AFP and BP families. Comparing the sequence logos of each of the three families, the overall higher conservation of the amino acids of the mature part of the BP family members stands out (Supplementary Fig. 5). Additionally, the Kex2-like protease cleavage site sequence LXXR for posttranslational processing is conserved in the AFP (LDAR) and the BP family (LEAR) but cannot be observed in the NFAP2 family [56, 57].

**Fig. 3:**
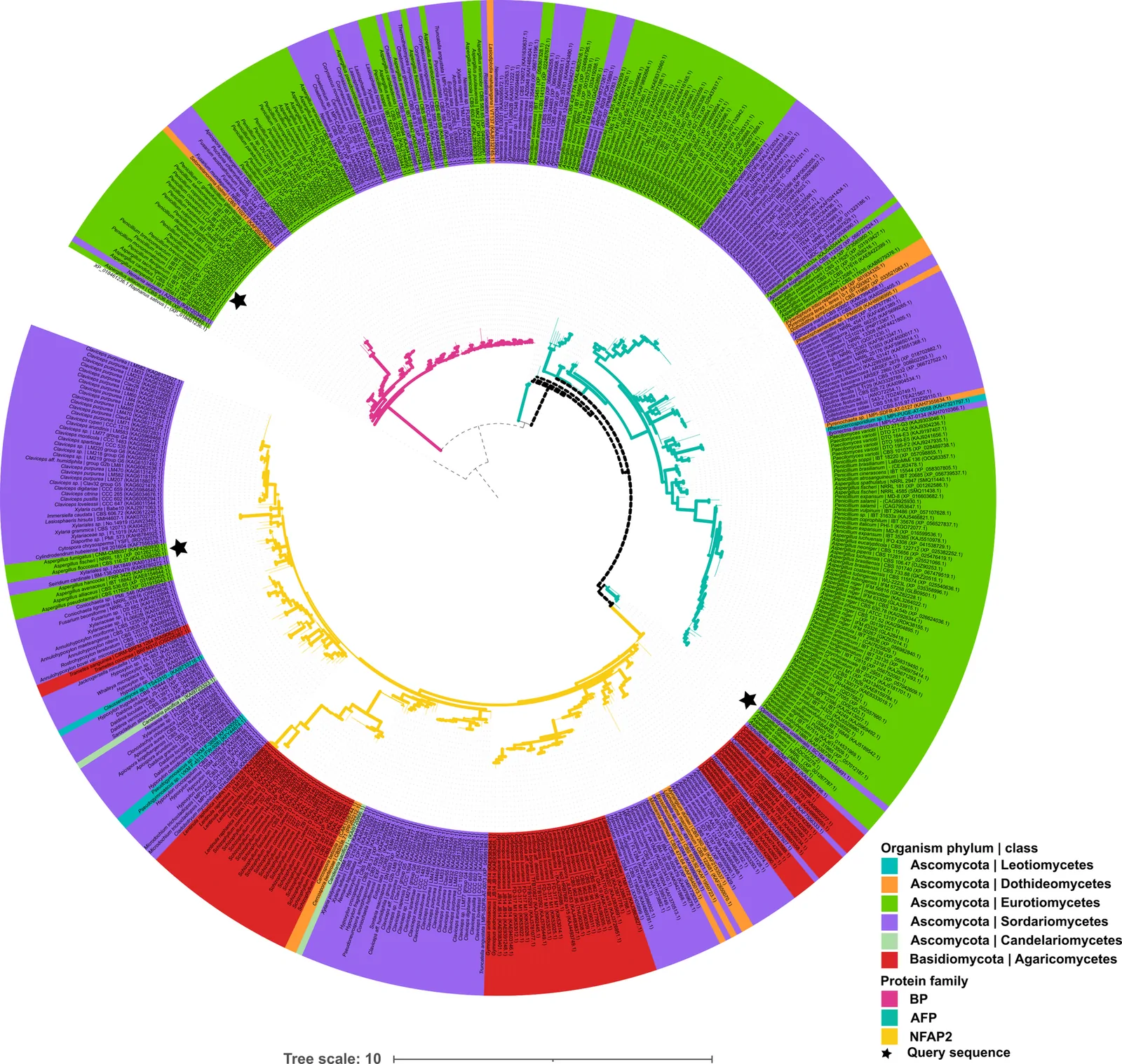
Phylogenetic analysis of mature antifungal protein homologues identified by PSI-BLAST in the NCBI non-redundant database. Using query sequences (AgAFP, PbBP, NFAP2) homologous proteins were determined using the PSI-BLAST tool in the NCBI non-redundant database. Proteins not meeting pre-defined criteria (Table 1) were excluded from further PSSM construction. This resulted in 165 AFP, 102 BP, and 219 NFAP2 family members. Next, putative amino acid sequences of mature proteins were aligned using the MAFFT algorithm, and the phylogenetic tree was generated with IQTree through a rapid bootstrap analysis consisting of 1,000 iterations. Outgroup rooting was performed using defensin-like protein 2 from *R. sativus*. The resulting tree was visualised in iTOL, where cladogram line thickness corresponds to increasing bootstrap support. Encoding organism names are presented alongside strain numbers and NCBI accession numbers in brackets

To further elucidate the evolution of the AFP, BP, and NFAP2 families, a phylogenetic analysis of the (putative) members retrieved by PSI-BLAST was performed for both the precursor proteins and the putative mature proteins (Supplementary Fig. 6 and Fig. 3). Both phylogenetic trees were build using the maximum-likelihood method with a bootstrap of 1,000 followed by outgroup rooting with the radish plant defensin RsAFP2 from *R. sativus*.

In both trees, a neat separation of the AFP, the BP, and the NFAP2 family is visible. Two major speciation events could have led to the three families. While the first separated the BP-like clade from the AFP-NFAP2-like clade, a second speciation led to the differentiation of AFP-like and NFAP2-like proteins. Therefore, members of the AFP family might be the origin of the currently known three families within the BPF. This could have happened based on gene duplication and subsequent change of function. A comparison of the two phylogenetic trees reveals that the mature protein and the pre-pro-protein have evolved at different rates, suggesting distinct evolutionary dynamics between the signal sequence and the mature protein.

Consistent with earlier studies, subgroups within the three families can be observed [16, 54]. For example, in the AFP family, proteins with six conserved cysteines can be distinguished from proteins with eight conserved cysteine residues (Supplementary Fig. 1). Also, the C-termini of AFP members show sequence and length variations which are shared among putative subgroups. In contrast, BP family members show a conserved cysteine pattern within their mature proteins, including all eight cysteines, and only a small group shows variations in the C-terminus (Supplementary Fig. 1). Members of the NFAP2 family can also be further divided regarding the C-terminus with respect to sequence and length variations (Supplementary Fig. 1). Furthermore, the pre-pro region of the proteins would also allow further subcategorisation with respect to length and sequence variations. Interestingly, variations in the pre-pro-regions do not correlate with the ones of the C-termini. However, despite the diversity within each of the three families, which is more pronounced in both the AFP and NFAP2 families compared to the BP family, and despite some AFP members having eight cysteines which is more similar to BP family members, and although the cysteine pattern of NFAP2-like proteins is in parts more similar to the cysteine pattern of AFP family members with eight cysteines, all 486 proteins were clearly assigned to their corresponding family. This emphasises the presence of clear and conserved differences within the overall primary structures of the three families. In summary, the BPF superfamily can be divided into three families (AFP, BP, NFAP2), which each can be divided into further subgroups depending on the region or characteristic of interest.

Within the presented dataset, the organism *Apiospora kogelbergensis* strain CBS 113332 was found to encode members of all three families, that is, next to one BP and one NFAP2 family member, two AFP family members, which appear on distinct branches (Supplementary Fig. 7). Only two other organisms encode members of all three BPF families: *Aspergillus fischeri* strain NRRL 181 and *Aspergillus hancockii* strain FRR 3425 (Supplementary File 1). Other organisms encode multiple AFP members (e.g., *Aspergillus chevalieri* strain M1, *Fusarium austroamericanum* strain NRRL 2903, *Penicillium salamii*), BP members (e.g., *Aspergillus puulaauensis* strain MK2, *Penicillium coprophilum* strain IBT 35676, *Corynascus sepedonium* strain ATCC 9787) or NFAP2 members (e.g., *Daedaleopsis nitida* strain CIRM-BRFM 1802, *Candelaria pacifica*, and multiple strains of *Claviceps purpurea*). Most strains, 90.0% (325), encode only one member of the three BPF families, whereas 9.1% (33) encode members of two, and 0.8% (3) encode members of all three BPF families (Supplementary Fig. 8). The parallel low occurrence of AFP and NFAP2 family members (only two cases) could hint towards a more similar function between AFP and NFAP2 compared to AFP and BP. Thus, it would be sufficient to own either one AFP or one NFAP2 family member. However, the clear diverge of the phylogenetic timeline and the variations of structural features within the protein primary structures suggest that members of the BPF superfamily serve maybe related but not identical purposes.

### Horizontal gene transfer might have distributed BPF-members among fungi

**Fig. 4:**
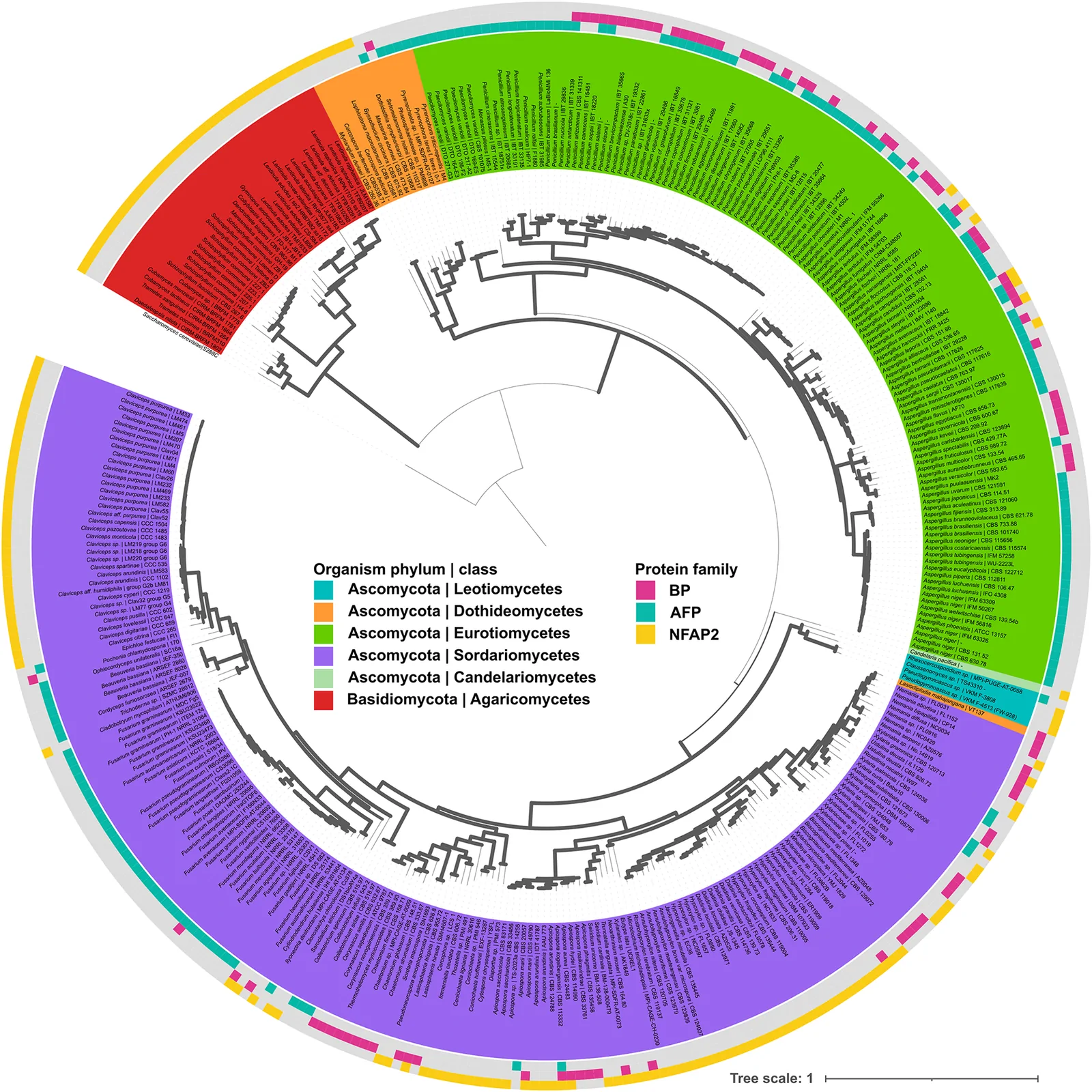
Phylogenetic analysis of 354 organisms encoding (putative) members of the BPF superfamily. Genome assemblies from NCBI were analysed using the UFCG pipeline by extracting 61 core genes, which were aligned and concatenated. The tree was build using the FastTree module and visualised using iTOL. *S. cerevisiae* genome assembly was used for outgroup rooting. Organisms not included due to lack of any genome assembly: *A. giganteus* and *A. spathulatus.* Outer rings show which organism encodes which protein family

To gain a comprehensive understanding of the evolution of the three protein families, their distribution among various fungal genera and their taxonomic relationships were examined. The UFCG pipeline was used to evolutionary analyse the fungal genome assemblies retrieved from the NCBI database. The trees were outgroup rooted using the yeast *Saccharomyces cerevisiae*. First, the fungi encoding BPF superfamily members were analysed (Fig. 4). The fungal classes are distinctly separated and the spread of the three protein families is patchy, which is in contrast to the phylogenetic analysis of the proteins (Fig. 3), indicating phylogenetic incongruence.

**Fig. 5:**
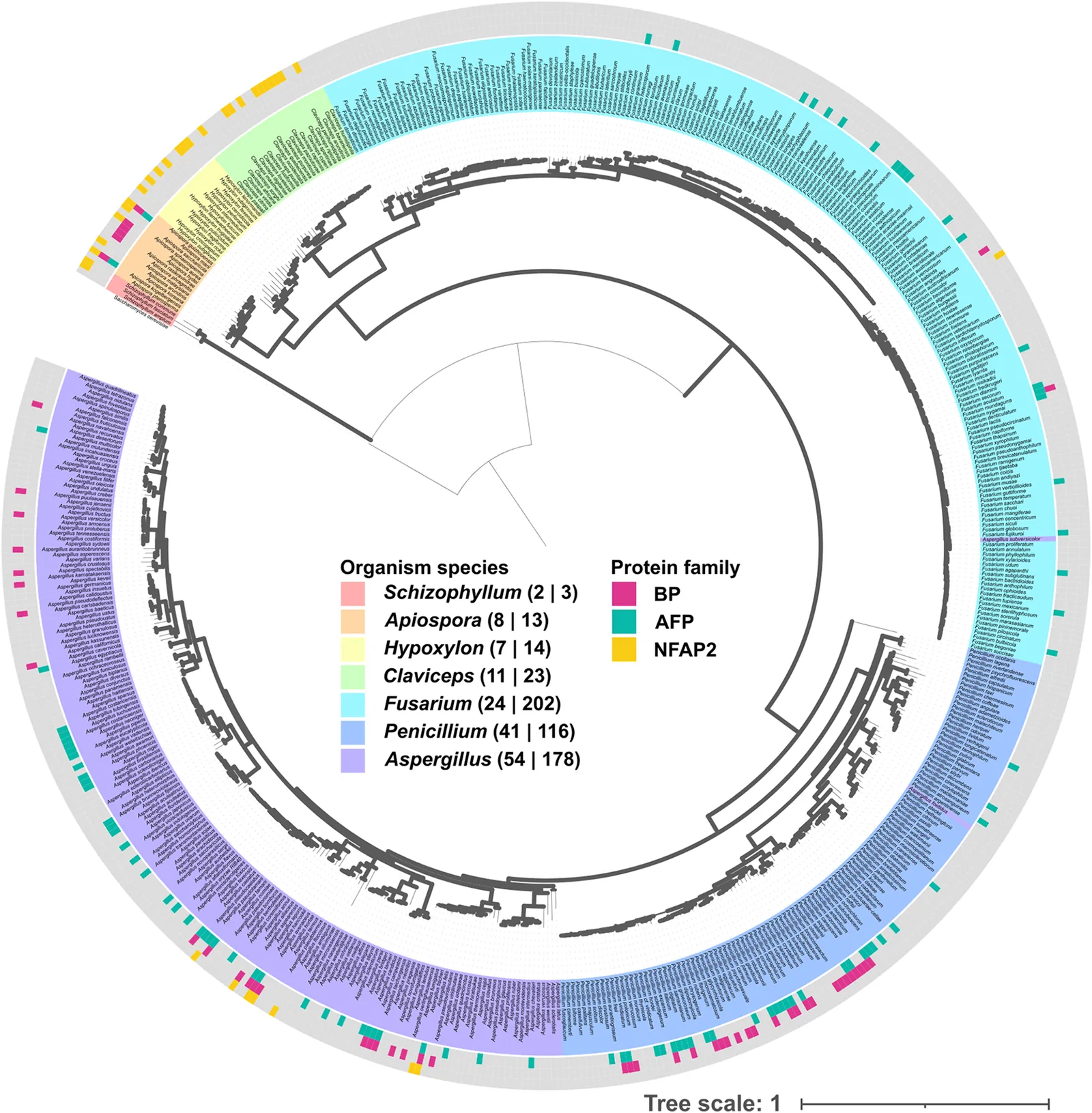
Phylogenetic species analysis of the main BPF encoding genera. Genomes were retrieved from NCBI and phylogenetic tree was constructed using the FastTree option within the UFCG pipeline by extracting 61 core genes, which were aligned and concatenated. The following organisms are excluded due to unavailable genomes: *A. giganteus* and *A. spathulatus*. The tree is outgroup rooted to the genome of *S. cerevisiae* and visualised using iTOL. Number of species encoding an BPF superfamily member, and number of reference genomes are given in brackets and separated by an |

Additionally, the genera encoding most of the BPF superfamily (Supplementary File 8), including *Aspergillus*: 18.3%, *Penicillium*: 15.8%, *Claviceps*: 10.1%, *Fusarium*: 7.6%, *Hypoxylon*: 4.3%, *Apiospora*: 3.9%, and *Schizophyllum*: 3.9%, were analysed on the species level to investigate the taxonomic distribution of AFP, BP and NFAP2 family members. As expected, the seven genera are clearly separated. The separation of one *Aspergillus* species (*A. subversicolor*) from the others in Fig. 5 is likely due to computational errors from the maximum likelihood method (FastTree), perhaps as consequence of a problem regarding genome quality, rather than true phylogenetic differences. However, the strain was not excluded to show the full set of available encoding fungi. Three genera (*Hypoxylon, Schizophyllum,* and *Claviceps*) exclusively encode members of the NFAP2 family. In contrast, *Penicillium* encodes only AFP and BP family members, while the genus *Fusarium* primarily encodes AFP family proteins. Within the genus *Penicillium* and *Fusarium*, evolutionary younger species encode more BPF superfamily members than older species. This is in contrast to the genus *Aspergillus*, which seems to be limited to either an AFP or BP family member after time. Additionally, BP encoding species show up later than AFP encoding species in all genera. This depicts a complicated evolutionary background, which could include genetic exchange between species or environmental adaptation. The phylogenetic incongruence in combination with the restricted taxonomic distribution visible in Fig. 5, suggests the possibility of a horizontal gene transfer of BPF between fungal species.

### Tertiary structures of BPF superfamily members differ from non-fungal small cysteine stabilised antifungal proteins

**Fig. 6:**
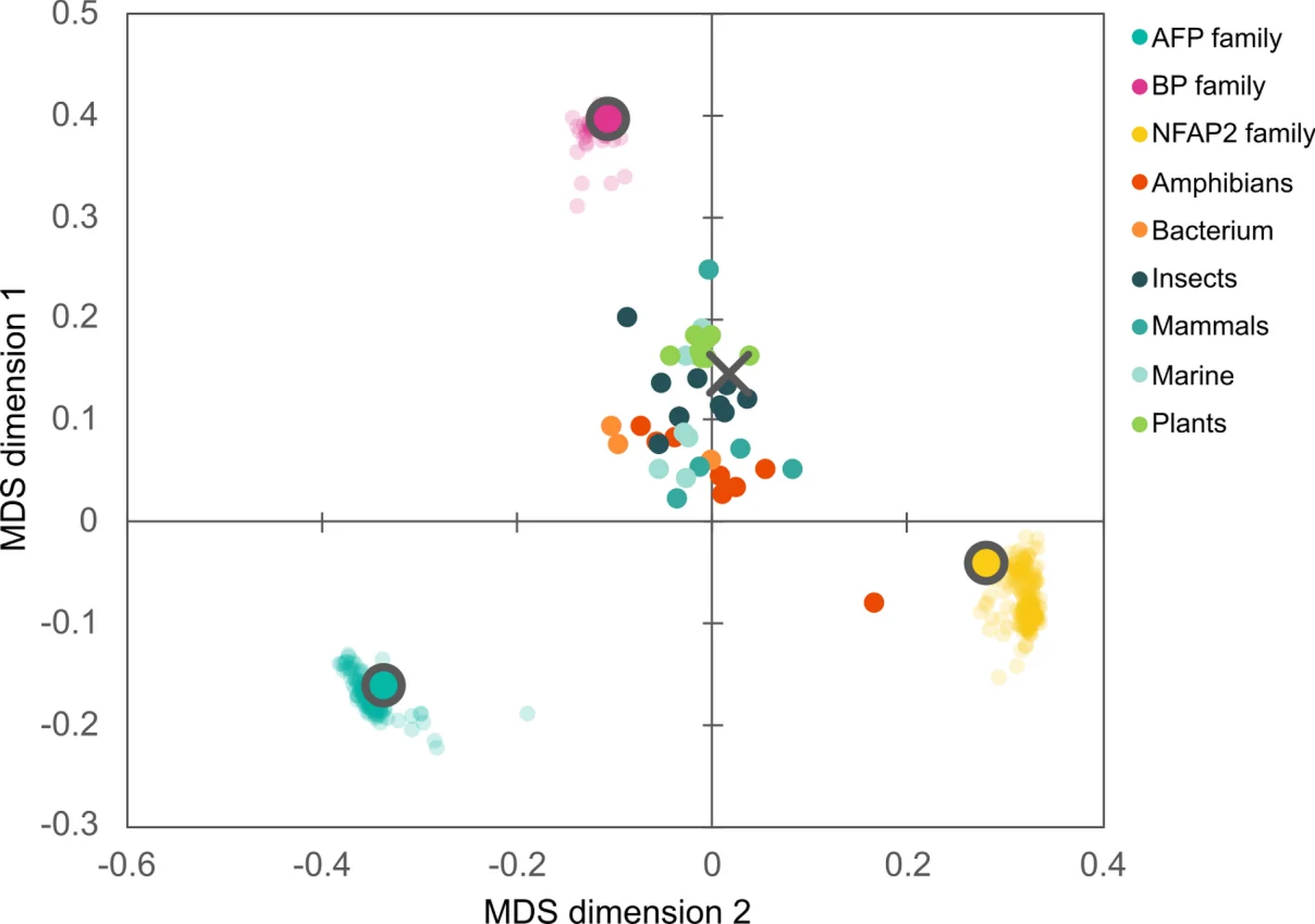
Tertiary structure comparison of antifungal proteins. Tertiary structures were compared using the USalign pipeline followed by distance (1-TMscore) conversion into two-dimensional coordinates using classical multidimensional scaling. Tertiary structures of all (putative) AFP, BP, and NFAP2 family members and of small (≤ 150 amino acids) cysteine stabilised antifungal proteins of different groups. Query proteins are indicated with a grey outlined circle and the reference structure of *R. sativus* RsAFP2 is denoted with an x

The function of a protein depends predominantly on its tertiary structure. Therefore, comparing primary structures and their phylogenetic relationships alone is not sufficient to draw conclusions on protein function. Hence, tertiary structure comparison was used to yield new insights into potential functions of BPF. The members of the AFP, BP, and NFAP2 families all cluster within their respective families (Fig. 6). Hence, the clear separation might be indicative of distinct functional difference among AFP, BP, and NFAP2 families.

To put the results of the structural analysis into a broader biological context, small cysteine containing antifungal proteins from other organism groups were compared with the experimentally determined (if available) or predicted tertiary structures of all BPF superfamily members. Using all BPF members brings the advantage of comparing the overarching protein tertiary structure of each family with the ones of non-fungal antifungal proteins. Interestingly, the three BPF families clearly distinguish from each other and the investigated antifungal proteins from other organisms. Hence, not only based on primary phylogenetic comparison but also on tertiary structural comparison, the AFP, BP, and NFAP2 families appear as distinct families of small, cysteine-rich antimicrobial proteins. Furthermore, the tertiary structure similarity of the proteins of other organisms indicates a structural background at whose edges the BPF families might have evolved and specialised in their specific structure and therefore function.

## Discussion

### Evolution of the BPF superfamily

The relative categorisation of members of AFP, BP, or NFAP2 family members is discussed controversially in available literature describing them, e.g., as different classes, clades, subclades, families, or even as superfamilies [5, 15, 16, 54]. Using phylogenetic approaches and structural analysis of these proteins which were exclusively encoded among fungi, we elucidated their evolution and distribution. According to the evolutionary relation between all investigated proteins, they occur as three distinct protein families (AFP, BP, and NFAP2 family distinguishable by their primary and tertiary structures and type, number, and position of γ-core motifs) and we suggest to merge them in one superfamily denoted as BPF. Superfamily traits shared among the three families are the exclusive distribution among fungi, the presence of a γ-core motif and signal peptide, a high cysteine amount and specific cysteine patterns, a basic and hydrophobic character, and the similar pre-pro-sequence pattern (see Table 1, Fig. 2, Table 3, and Supplementary Fig. 4). All verified members have been shown to exert antimicrobial activities which could be considered either. However, further experiments are required to evaluate if this is a common trait shared among all BPF members described in the current study. In accordance with other studies, further subcategorisation within the three families would be possible, depending on the considered region or characteristic (e.g., number of cysteines, C-terminus variation). For example, Garrigues et al. have described the three classes A, B, and C, where classes A and B belong to the AFP family and class C to the BP family [54]. Another way of subcategorisation was presented by Sonderegger et al. who have defined subclades depending on the charge of the γ-core motif [16]. However, based on the large number of peptides included in our study, we did not focus on a subcategorisation in more detail. Interested readers might be referred to corresponding literature.

According to this study, the BPF superfamily has at least 486 putative members. This number is expected to increase further the more fungal genomes become sequenced and annotated revealing more protein sequences. Even more important, the BPF superfamily distributes among the evolutionary distinct divisions of Ascomycetes and Basidiomycetes. Since both divisions diverged during evolution from Dikarya, a common ancestor antifungal protein might be the origin of the BPF superfamily. However, their evolution might have begun also after divergence from Dikarya presumably driven by HGT. Points supporting this hypothesis include (1) a high sequence similarity, shown by the BLAST algorithm finding homologous proteins, (2) an irregular distribution of the proteins within one genus, which was shown in Fig. 5, and (3) phylogenetic incongruence, which becomes clearly apparent when comparing the phylogenetic trees for protein homologues and protein encoding fungi of Figs. 3 and 4 [58, 59].

One main driving force of evolution is gene duplication, followed by mutations which either lead to loss of function, neofunctionalisation, subfunctionalisation, or dosage selection [60, 61]. The different clade sizes of the protein analysis and the occurrence of two members of the same BPF family within one strain, are strong indicators that gene duplication followed by loss or gain of function could have played a major role in BPF superfamily development [62]. This was also exemplified using an unrooted tree, which simplifies the detection of duplicates within one family (Supplementary Fig. 7). In addition, the analysis of the tertiary structure, with the three families placed at different sites and with clear distance to other non-fungal antifungal proteins gathering closely together between them, might point towards a divergent evolution after HGT of a small, cysteine rich blueprint (Fig. 6). However, further research is required to elucidate the hypothesis of HGT and to provide genomic context analyses, e.g., by analysis of the GC content, synteny, codon usage, etc. Thus, the distribution of BPF members among fungi by HGT is a possible but so far unproven hypothesis.

Interestingly, to our best knowledge only one NFAP2 member was described before this study, including investigation of its antimicrobial activity [22]. The studies of Sonderegger et al. (2018) and more recently of Gai et al. (2025) already indicated that at least 15 homologues exist in the available databases [16, 63]. Based on the presented data, the NFAP2 family is currently the largest group within the BPF superfamily. Considering 41.2% of *Claviceps* spp. and 28.4% of all NFAP2 encoding strains have two or more NFAP2 copies (compared to AFP: 7.1%, BP: 9.9%), it might be presumed multiple versions of the same protein give the organism an advantage, e.g., in form of increased functional diversity. Since only protein and not genomic data were investigated in this study, it remains to be hypothetical, if multiple proteins are originating from a single gene or if the gene was duplicated.

A closer examination of the pre-pro-sequence in the sequence logo of each BPF family reveals, that the Kex2-like cleavage site (LXXR) does not exist within the NFAP2 family. This suggests either no further processing of NFAP2 family members compared to the other two families or a different processing mechanism using a different enzyme. Additionally, the location of the motif differs in the BP and the AFP family. The cleavage site is directly at the start of the mature form in the BP family, but a few amino acids separated from the mature protein in members of the AFP family. Two fungi (*A. giganteus* and *Epichloë festucae*) have been shown to produce a so-called large form which exerts a significantly reduced antifungal activity compared to the mature form of the proteins [64, 65]. Both protein members belong to the AFP family. Since most AFP family members possess the LXXR motif we suggest that they can also express a large form, presumably as regulatory means. This indicates that each protein family might have unique ways of posttranslational processing, which might also be related to possible diverse functions.

### Can BPF members be considered as fungal secondary metabolites?

According to Keller et al., secondary metabolites (i) have a low molecular weight, (ii) are bioactive, (iii) their genes are often found in gene clusters, (iv) their function is related to specific life cycle stages (often morphological differentiation), (v) they are not primarily relevant for survival, and (vi) they have a restricted taxonomic distribution [66]. The currently distinguished classes of secondary metabolites include polyketides, alkaloids, non-ribosomal peptides, terpenoids, and RiPPs (ribosomally synthesised and post-translational modified peptides). BPF members are ribosomally synthesized and encoded from single genes (shown for the verified members). However, they also must undergo specific modifications to mature. For example, in case of AgAFP, there exist at least three native forms of the peptide, which comprise a large form, a mature form, and a short form each showing different antifungal activities [52, 65]. Further forms of the peptide must exist and be processed to mature from the pre-pro-protein to the three proteins. According to the sequence logo, the pre-pro-structure is conserved throughout the AFP-family (Supplementary Fig. 5). Note, the processing mechanism and machinery for these proteins are not yet fully elucidated and require further research. Secondary metabolite maturation occurs in several steps using additional enzymes which results in product variation. However, up to now, there is no direct evidence supporting the presence of a targeted enzymatic processing cascade or the presence of different specific processing enzymes involved in the generation of AFP variants. Thus, the observed protein variants might be the consequence of a natural variability in proteolytic processing of secreted proteins. Consequently, it is not yet possible to unambigously categorize BPF as RiPPs.

As discussed above, our results show a restricted taxonomic distribution of BPF family members. Furthermore, at least all verified BPF members show bioactivity in form of antimicrobial activity and, for some family members, further characteristics can be observed, like involvement in asexual development and/or that they are not required for colony survival [2, 4, 53, 54, 67]. Note, to date, only a few of the 486 BPF superfamily members have been investigated in the laboratory. Furthermore, only a few of these have been investigated for properties beyond their antimicrobial character. Several examples from all three protein families show a late expression of proteins (AnAFP, AFP, AgBP, NFAP, NFAP2, PAF, PAFB, PAFC, PeAfpA, PbBP) in combination with nutrient limitation or were isolated from several days’ old cultures, i.e., after the exponential growth phase [21, 22, 51, 68, 69]. However, some examples exist which do not align with this observation. For example, whereas PeAfpA (AFP member derived from *Penicillium expansum*) is expressed under nutrient limitation but not expressed in nutrient rich medium, the expression of PeAfpB (AFP member) and PeAfpC (BP member) both also derived from *P. expansum* could not be observed under nutrient rich or depleted conditions [68]. In addition, PAFB derived from *P. chrysogenum* is expressed under nutrient excess (thus, before stationary phase) but only weekly under nutrient depletion [70]. However, it is noticeable, that in the latter and controversial examples proteins were derived from the same host. One of the genes is aligning with late expression and the other/s is/are not. If considering gene duplication and HGT as origin for the increased number of BPF in the same host independent neo- or subfunctionalisation would allow modification of expression regulation. Furthermore, the example of AnAFP from *A. niger* shows how complex expression regulation can be. The *anafp* gene has been recently demonstrated to be under a tight temporal and spatial expression control leading to a controversial expression pattern within the hyphae of its producing strain [2]. In summary, the characteristic of fungal secondary metabolites, that their function is related to specific life cycle stages (often morphological differentiation) cannot be assigned unambiguously for BPF. In particular, there is currently no biochemical or genetic evidence to support a direct role for BPF in secondary metabolic pathways.

To the best of our knowledge, no study has yet shown that BPF member genes are organised in gene clusters. However, per definition, the minimum number of genes in a gene cluster is two [71]. Furthermore, there are examples for secondary metabolite gene clusters, where corresponding genes are distributed over multiple loci or are “unclustered” [66, 72–74]. As already mentioned above, there is no study demonstrating that specific processing enzymes are involved in maturation of any BPF member. Further research is needed to explore that.

The last attribute which must be discussed is the “low molecular weight”. Regarding the size of “common” secondary metabolites, like polyketides, alkaloids, terpenoids, etc., they are generally smaller than BPF. However, the precursor molecule of the secondary metabolite nisin (which is a RiPP) comprises 57 amino acids and the final molecule more than 34 amino acids [75]. According to the study of Mohanta et al. the average molecular weight of a fungal proteome is ca. 51 kDa [76]. The authors have analysed 689 fungal species and found that the highest average molecular weight of fungal proteins was ca. 71 kDa and the lowest ca. 20 kDa. Moreover, the highest average number of amino acids per protein sequence was found in Ascomycetes and Basidiomycetes (ca. 475 and 446, respectively). Thus, with ca. 60 residues for the mature forms, BPF are merely two times larger than nisin and regarding the length of the pre-pro-proteins (ca. 100 amino acids) they are ca. 5 times smaller than the average fungal protein in their phyla and might still be considered small. In summary, there are some characteristics (i, ii, v, and vi) which align with common features of secondary metabolites according to Keller et al. (see above). However, other characteristics defined by Keller et al. (iii, iv) and also the processing relevant for RiPP assignment are inconclusive.

Considering the BPF superfamily as secondary metabolites could extend the perspective on these proteins, and the information about other secondary metabolites can be leveraged to pinpoint the different endogenous function of their members. Until further evidence has been provided and since not all of the characteristics defined for secondary metabolites can be unambiguously assigned, we suggest that members of the BPF superfamily could be considered as “secondary metabolite like” proteins.

Interestingly, BPF members are present in several genera of fungi with economic relevance, e.g., *Penicillium, Aspergillus, Trichoderma, Thermothelomyces, Lentinula, Trametes, Schizophyllum*, [77, 78]. The potential secondary metabolite-like character of BPF together with bioactivities related to carbon metabolism, autophagy, etc., reported for some BPF members. [2, 3], might interfere with production rates and should be considered during genetic and metabolic optimisation. However, since the presented data also contains pathogenic fungi as *Fusarium graminearum*, *Beauveria bassiana*, and *Claviceps purpurea*, the expression of BPF superfamily members during virulence is another point to be considered in future.

### Structure and function of BPF members

When chitin affinity chromatography has been applied AgBP was never isolated before. Thus, chitin binding can be excluded as part of its antifungal mechanism. In comparison to AgAFP, AgBP exerts a similar activity spectrum towards the same set of tested microorganisms but AgAFP showed lower MIC values, e.g., 1 µg mL^−1^ for *A. niger* and for *F. sporotrichioides* [53]. However, the set of tested microorganisms in this study comprising representatives of Gram-positive and Gram-negative bacteria, yeast, and filamentous fungi can provide only a preliminary overview. To further evaluate the antimicrobial character of AgBP, more microorganisms and, also, different experimental conditions must be tested. For example, *Paecylomyces variotii* (also known as *Byssochlamys spectabilis*) has been recently described as a very susceptible target strain towards the BP family member AfAfpC from *A. fischeri* [32].

Since, AgBP was expressed at a relative late cultivation time (72 h) beyond the exponential phase (Supplementary Fig. 9B) and in absence of microbial competitors (axenic *A. giganteus* culture), a further biological role of AgBP might be conceivable. Interestingly, bubble protein-like proteins show a clearly higher primary structure conservation compared to AFP and NFAP2 families which is consistent with other studies [32, 54]. As shown for *A. giganteus*, all of the four tested strains contained more primary structure deviations in the *agafp* gene than in the *agbp* gene. Whereas the AgBP was 100% conserved, several mutations have been observed in case of AgAFP (Supplementary File 7). According to the emergence of antimicrobial resistance the accumulation of mutations in corresponding antimicrobial protein genes would make sense to allow adaption.

The sequence logos (Supplementary Fig. 5) of all putative BPF members highlight the high conservation of cysteines, which is probably related to the structure that becomes stabilised and is expected for secreted proteins. Also, basic residues are conserved in all three BPF families which is also expected for proteins with antimicrobial character [14]. However, whereas BP-like proteins are characterised by arginine residues, AFP-like proteins and NFAP2-like proteins are characterised by lysines. Since approximately 25% of the amino acid residues of AFP-like proteins and NFAP2-like proteins are highly conserved, these residues might solely be required to adopt the corresponding protein folds. The remaining 75% of residues are very likely freely exchangeable. Hence, both families represent extraordinary template structures for targeted structural design. Interestingly, there is one region in NFAP2 family proteins, ACNCPNNC, which is highly conserved compared to the remaining primary structure and might therefore be linked to the proteins’ fold and/or function. The overall higher conservation of BP-like proteins might suggest a reduced structural flexibility for adoption of the knottin-fold and/or hint to a larger dependence of the primary structure on the proteins’ biological function. Consequently, given their presumably limited design flexibility, BP-like proteins appear less suitable for use as a structural template for the development of antimicrobial peptides in direct comparison to AFP- and NFAP2-like proteins.

All 486 members of the BPF superfamily were exclusively identified in fungi. Interestingly, there is no yeast strain among the identified fungi which encodes a BPF homologue. This might indicate that the function of BPF members is related to specific conditions which are only experienced by Ascomycetes and Basidiomycetes. This might comprise the defense against specific competitors of the same ecological niche but also the communication with “commensals” or a host-related tasks for self-regulation/organisation.

As has been discussed above, the expression regulation differs among members of the BPF superfamily. Even though the various effectors and signalling pathways involved in regulation have not yet been fully elucidated, the reported differences in gene regulation and expression in response to environmental conditions may indicate functional diversification among BPF members. Historically, due to a biased search approach targeting compounds with antimicrobial character the functions of the first identified BPF members were categorised accordingly. However, an increasing number of studies report on other biological functions not related to a defense function [2, 3, 79, 80]. Furthermore, the tertiary structures of BPF members strongly differ from non-fungal antifungal proteins (Fig. 6), further indicating different functions. Based on the discussion of secondary metabolite-like characteristics in BPF many functions are conceivable.

## Conclusion

In summary, this study emphasises the growing reservoir of a superfamily of bioactive proteins of fungal origin which is spanning two divisions of fungi: Ascomycetes and Basidiomycetes. We hypothesise that BPF superfamily members were presumably spread by HGT and gene duplication probably paralleled by subsequent neo- or subfunctionalisation, that can be assumed to drove further development. Although BPF have been described as antifungal compounds there are indications that they exert other functions with a similar or perhaps even higher relevance. It is important to interpret these findings within the context of cultivation conditions. Such conditions may subject organisms to environmental selective pressures and observed gene duplications could reflect short-term adaptation responses [60, 61]. However, these can lead to later retainment of the gene due to fitness advantages [60, 61]. Nevertheless, considering BPF as secondary metabolite like proteins rather than solely antifungal proteins, would enable a shift of perception towards other biological functions. The presence of BPF members in several genera of fungi with economic relevance reinforces the need for further investigation into their potential impact on fungal biology and biotechnology.

## Supplementary Information


Supplementary file 1.
Supplementary file 2.
Supplementary file 3.
Supplementary file 4.
Supplementary file 5.
Supplementary file 6.
Supplementary file 7.
Supplementary file 8.
Supplementary file 9.
Supplementary file 10.
Supplementary file 11.
Supplementary file 12.
Supplementary file 13.
Supplementary file 14.
Supplementary file 15.
Supplementary file 16.
Supplementary file 17.
Supplementary file 18.


## Data Availability

The datasets supporting the conclusions of this article are included within the article and its additional files.

## Abbreviations

AFP: Antifungal protein
BPF: Bioactive proteins from fungi
BP: Bubble protein
NFAP2: Neosatorya fischeri Antifungal Protein 2
HGT: Horizontal gene transfer
