# Supplementary material for: A superfamily of bioactive proteins from fungi - are these secondary metabolites?
Source: Fungal Biol Biotechnol. 2026 Aug 29;13:15. doi: 10.1186/s40694-026-00219-x (PMC13525654; doi:10.1186/s40694-026-00219-x)
Supplement: Supplementary file 1 [file 40694_2026_219_MOESM18_ESM.docx]

Legends to Supplementary Figures to manuscript

**A superfamily of bioactive proteins from fungi – Are these secondary metabolites?**

**Supplementary Figure 1 | Multiple sequence alignment of the BPF superfamily.** Multiple sequence alignment was performed and visualised using the MAFFT algorithm and Jalview, respectively. Red squares indicate the position of the γ-core motif and beginning of putative mature peptides are highlighted in blue. Query sequences for AFP (*A. giganteus*), BP (*P. brevicompactum*), and NFAP2 (*A. fischeri*) family members are labelled by a pink frame.

**Supplementary Figure 2 | *Aspergillus giganteus* bubble protein primary and tertiary structure.** Primary structure was determined using trypsin digestion and LC-MS/MS. Tertiary structure was generated using AlphaFold und visualised with ChimeraX. Disulfide forming cysteines are highlighted in yellow and the γ-core motif in blue.

**Supplementary Figure 3 | Microtiter susceptibility assays of filamentous fungi and yeast.** OD_600_ was measured after organisms were grown in YPD for 48 hours at 30 °C in presence of different concentrations of AgBP and normalized to controls without AgBP. Median values and standard deviations from quintuplicate measurements are shown, calculated using the Microsoft 365 Excel STDEVP function. Representative results from two independent experiments.

**Supplementary Figure 4 | Quantitative analysis of primary structure characteristics of antifungal protein homologues identified by PSI-BLAST in the NCBI nr database.** Visualisation and statistical analysis disclosed a high amount of statistically significant differences in primary structure characteristics based on Students t-test. To indicate high similarity, p-value > 0.05 is highlighted with an asterisk.

**Supplementary Figure 5 | Sequence logo of the members of each BPF family.** Multiple sequence alignment was performed and sequence logos generated using the MAFFT algorithm and WebLogo3 for published putative members of the AFP (top), the BP (middle), and the NFAP2 (bottom) family [28-31]. Colour scheme according to chemical properties: hydrophobic (black), basic (blue), neutral (pink), acidic (yellow), and polar (green).

**Supplementary Figure 6 | Phylogenetic analysis of antifungal protein homolog precursors identified by PSI-BLAST in the NCBI nr database.** Using query sequences (AgAFP, PbBP, NFAP2) homologous proteins were determined using the PSI-BLAST tool in the NCBI non-redundant database. Proteins not meeting pre-defined criteria (Table 1) were excluded from further PSSM construction. This resulted in 165 AFP, 102 BP, and 219 NFAP2 family members. Next, precursor sequences were aligned using the MAFFT algorithm, and the phylogenetic tree was generated with IQTree through a rapid bootstrap analysis consisting of 1,000 iterations. Outgroup rooting was performed using defensin-like protein 2 from *R. sativus*. The resulting tree was visualised in iTOL, where cladogram line thickness corresponds to increasing bootstrap support. Encoding organism names are presented alongside strain numbers and NCBI accession numbers in brackets.

**Supplementary Figure 7 | Phylogenetic analysis of the members of the BPF superfamily encoded by *Apiospora kogelbergensis* strain CBS 113332.** Using query sequences (AgAFP, PbBP, NFAP2) homologous proteins were determined using the PSI-BLAST tool in the NCBI non-redundant database. Next, precursor sequences were aligned using the MAFFT algorithm, and the phylogenetic tree was generated with IQTree through a rapid bootstrap analysis consisting of 1,000 iterations. The unrooted tree was visualised in iTOL, where cladogram line thickness corresponds to increasing bootstrap support. Proteins encoded by *A. kogelbergensis* strain CBS 113332 are indicated with circles.

**Supplementary Figure 8 | Distribution of strain counts by each BPF family.** Venn diagram was used to visualise the number of strains encoding one, two, or three different BPF family members.

**Supplementary Figure 9 | Purification of AgBP from *A. giganteus*. A)** SDS-PAGE gel showing a single-peak purified AgAFP control sample and a 20% acetonitrile elution fraction from hydrophobic interaction chromatography (Sep-Pak, tC18 column) of a 96 h incubated *A. giganteus* cultivation (shake flask, 30 °C, 150 rpm). Bands from PageRuler™ unstained protein ladder, low range are labelled. The 10 kDa band was to faint for detection. Dashed lines help to distinguish the different retentions times of the bands. **B)** SDS-PAGE gel showing 20% acetonitrile elution fractions from hydrophobic interaction chromatography (Sep-Pak, tC18 column) of a cultivation time series. Purification was done from *A. giganteus* cultures (shake flask, 30 °C, 150 rpm) after 24 h, 48 h, 72 h, or 96 h, respectively. Non-labelled gel pockets have been loaded with water control samples as in A. Bands from PageRuler™ unstained protein ladder, low range are labelled. The dashed line helps to distinguish the different retention times of the bands. Note, the defined protein band at ca. 15 kDa in the 48 h sample could neither be related to AgBP nor AgAFP. **C)** Cation-exchange chromatogram of a 20% acetonitrile elution fraction from hydrophobic interaction chromatography (Sep-Pak, tC18 column) of a 96 h incubated *A. giganteus* cultivation (shake flask, 30 °C, 150 rpm). The elution fraction was diluted 1:3 in phosphate buffered saline and 35 mL of the diluted sample have been loaded on the column (TOYOPEARL Sulfate-650F, 5 ml, SkillPak, Tosoh Bioscience) before a continuous gradient buffer exchange (2% min^-1^) from 50 mM NaOAc, pH 5 to 1.5 M NaCl, 50 mM NaOAc, pH 5 was applied. Absorbance is shown for 280 nm, 260 nm, and 214 nm in different colors. The absorbance signal was recorded during the whole procedure, i.e., starting when the sample was loaded on the column. mAU: milli-Absorbance Units.
